# Identification of an N-terminal 27 kDa fragment of *Mycoplasma pneumoniae *P116 protein as specific immunogen in *M. pneumoniae *infections

**DOI:** 10.1186/1471-2334-10-350

**Published:** 2010-12-13

**Authors:** Irum Tabassum, Rama Chaudhry, Bishwanath Kumar Chourasia, Pawan Malhotra

**Affiliations:** 1 Department of Microbiology, All India Institute of Medical Sciences, New Delhi-110029, India; 2 Malaria Laboratory, International Centre for Genetic Engineering and Biotechnology, New Delhi-110067, India

## Abstract

**Background:**

*Mycoplasma pneumoniae *is an important cause of respiratory tract infection and is increasingly being associated with other diseases such as asthma and extra-pulmonary complications. Considerable cross-reactivity is known to exist between the whole cell antigens used in the commercial serological testing assays. Identification of specific antigens is important to eliminate the risk of cross-reactions among different related organisms. Adherence of *M. pneumoniae *to human epithelial cells is mediated through a well defined apical organelle to which a number of proteins such as P1, P30, P116 and HMW1-3 have been localized, and are being investigated for adhesion, gliding and immunodiagnostic purposes.

**Methods:**

A 609 bp fragment P116_(N-27), _corresponding to the N-terminal region of *M. pneumoniae *P116 gene was cloned and expressed. A C-terminal fragment P1_(C-40), _of P1 protein of *M. pneumoniae *was also expressed. Three IgM ELISA assays based on P116_(N-27), _P1_(C-40) _and (P116 _(N-27) _+ P1_(C-40)_) proteins were optimized and a detailed analysis comparing the reactivity of these proteins with a commercial kit was carried out. Comparative statistical analysis of these assays was performed with the SPSS version 15.0.

**Results:**

The expressed P116_(N-27) _protein was well recognized by the patient sera and was immunogenic in rabbit. P1_(C-40) _of *M. pneumoniae *was also immunogenic in rabbit. In comparison to the reference kit, which is reported to be 100% sensitive and 75% specific, ELISA assay based on purified P116_(N-27), _P1_(C-40) _and (P116_(N-27) _+ P1_(C-40)_) proteins showed 90.3%, 87.1% and 96.8% sensitivity and 87.0%, 87.1% and 90.3% specificity respectively. The p value for all the three assays was found to be < 0.001, and there was a good correlation and association between them.

**Conclusion:**

This study shows that an N-terminal fragment of P116 protein holds a promise for serodiagnosis of *M. pneumoniae *infection. The IgM ELISA assays based on the recombinant proteins seem to be suitable for the use in serodiagnosis of acute *M. pneumoniae *infections. The use of short recombinant fragments of P116 and P1 proteins as specific antigens may eliminate the risk of cross-reactions and help to develop a specific and sensitive immunodiagnostic assay for *M. pneumoniae *detection.

## Background

*M. pneumoniae *is among the most common causes of community-acquired respiratory tract infection [1]. *M. pneumoniae *causes upper and lower respiratory illness in all age groups and accounts for 3.3-40% of community-acquired pneumonia (CAP) cases worldwide with an attack rates ranging from 25-71% among closed populations. Approximately 25% of individuals infected with *M. pneumoniae *develop extra-pulmonary complications [2].

*M. pneumoniae *infection is frequently seen in the patients with respiratory illnesses [1]. Recently an increased association of *M. pneumoniae *has been reported in acute exacerbation of bronchial asthma and chronic obstructive pulmonary disease (COPD) [3-5], acute respiratory distress syndrome (ARDS) [6,7], polyarthritis [8], stroke [9], Guillain-Barre syndrome [10], coronary artery diseases (CAD) [11,12] and increased seroprevalence in HIV positive patients with respiratory infections [13]. Therefore, presence of *M. pneumoniae *needs to be considered in the differential diagnosis of various respiratory and non-respiratory infections as the pathogen respond well to antibiotics, such as tetracyclines, macrolides and quinolones [14].

Mycoplasmas are cell wall deficient, the smallest known self-replicating organisms. They possess a small genome (0.58-2.20 Mbp) [15]. *M. pneumoniae *possess an apical organelle that mediates adherence to the host epithelium. The cytadherence is a complex multifactorial process requiring a group of proteins such as P1(170 kDa), P30(30 kDa), P116(116 kDa), HMW1-3 and proteins A, B and C. These proteins cooperate structurally and functionally for adherence as well as for the gliding mobility [16]. Among these apical organelle associated proteins, P1 and P30 have been previously shown by us and others to elicit immunological responses in human and are also involved in binding to host epithelial receptors [17-21]. In addition to these proteins, a 116-kDa protein of *M. pneumoniae *was identified as a membrane protein in triton-X-114 soluble fraction of the pathogen and its gene was identified. P116 protein is encoded in an operon, consisting of a 3093 bp ORF(orf 1030, Mpn number 213) encoding a protein of a predicted molecular mass, 116 kDa and a 408 bp ORF(orf 135, Mpn number 212) that is predicted to code a 16 kDa protein (5'-16 kDa ORF-116 kDa ORF-3'). Northern blot analysis and RT-PCR amplification of the intergenic region established that the ORFs encoding the 16 kDa and 116 kDa proteins are transcribed as a single mRNA. Swenstrup and co-workers showed that the P116 protein is expressed on the surface of *M. pneumoniae *and a polyclonal antibody Pab(rP116) inhibited the adherence of *M. pneumoniae *to Hep-2 cells [22]. These results demonstrated the role of *M. pneumoniae *P116 in cytadherence, like the other two adhesins P1 [23] and P30 [24]. A couple of studies have demonstrated the immune responses to the P116 fragment [25]. Our group has been working on the identification and characterization of the possible immunodominant molecules of *M. pneumoniae *for their immunodiagnostic potential. In the present study, we expressed and purified an N-terminal fragment (203aa) of P116 protein and showed that the protein is well recognized by sera of *M. pneumoniae *infected Indian patients. We next compared the immunoreactivity of recombinant P116 and P1 proteins with that of a commercially available ELISA kit - the Serion ELISA classic IgM(Virion-Serion, GmbH, Germany). Our results show that a short N-terminal fragment (203aa) of P116 protein holds a promise for serodiagnosis of *M. pneumoniae *infection.

## Methods

### Study subjects and diagnosis

The study was carried out with the approval of the Institute's human & animal ethics committee (Ref. 51/7.1.2004/AIIMS). Blood Samples were collected over a period of two and a half years i.e Sep.05 through March 07 from 62 patients between 1 m to 70 yrs of age, admitted in the wards and intensive care units of AIIMS hospital. Each patient's medical history was recorded in a specific format and written informed consent was obtained.

Commercially available Serion ELISA classic IgM (Virion-Serion, GmbH, Germany) kit, which uses mixture of undefined *M. pneumoniae *antigens, was used as screening test as well as reference test for comparison. This kit was selected, because of its availability and its use in various studies [17,26,27].

### Bacterial strains and culture conditions

*M. pneumoniae *standard strain FH (NCTC 10119), was grown in Edward Hayflick medium containing PPLO basal broth with supplements including glucose(1%; Difco, USA) and phenol red (0.0002%) as indicator in glass tubes. The culture was grown at 37°C, aerobically until the colour of the medium changed (red-orange). Mycoplasma cells were harvested at this stage by centrifugation at 15,000 rpm for 15 min, washed twice with PBS (pH 7.2) and were stored at -70°C until use. To confirm that the colour change of culture medium is due to *M. pneumoniae *growth, the broth culture was plated on PPLO agar plates and the plates were incubated at 37°C in a CO_2 _incubator. The plates were examined microscopically once in five days with X10 magnification. The suspected colonies were stained with Diene's stain and checked under light microscopy. Further confirmation of the colonies was carried out by growth inhibition assay using *M. pneumoniae *polyclonal antisera (NCTC).

### PCR amplification and cloning of P116-609 bp fragment

To clone 609 bp fragment of *M. pneumoniae *P116 gene that codes for a protein of 203 amino acids, primers were designed using published sequence (accession no. Z71425) [28]. *M. pneumoniae *genomic DNA was extracted as per the method of Stauffer et al [29]. Following set of primers with underlined introduced restriction sites (Microsynth, Switzerland) were used for the gene amplification:

The F1 was positioned at 786 nt whereas R1 at 1375 nt within the gene coding for P116 protein. PCR amplification was performed in a reaction volume of 50 μl containing 1× PCR buffer(100 mM Tris-HCl, pH 9.0, 500 mM KCl, 15 mM MgCl_2 _and 0.1% gelatin), 200 μm dNTP's, 20 pmol of each primer, 1U Taq Polymerase (5U/μl, MBI, Fermentas) and template DNA (50 ng) in a GeneAMP PCR system 9700 (Applied Biosystems, Switzerland). The PCR conditions were-initial denaturation at 94°C for 5 min followed by 30 cycles of amplification (each of 94°C for 30 s, 46°C for 30 s and 72°C for 1 min) with final extension at 72°C for 5 min. The PCR product was analysed by 1% agarose gel electrophoresis in 0.5% Tris-borate EDTA buffer and purified by gel extraction kit (Qiagen, Germany).

### Cloning and Expression of N-terminal fragment of P116 protein of *M. pneumoniae *in a prokaryotic expression system

The purified P116 gene fragment was ligated into pGEMT-easy vector and the ligation mixture was used to transform DH5-α *E. coli *cells. Colonies were selected on Luria Bertani (LB) agar plates containing 100 μg/ml of ampicillin, 20 mg of X-gal and 200 mg/ml of IPTG (Sigma-Aldrich, USA). The selected white colonies were further analyzed for the presence of P116 gene fragment. Recombinant plasmids were extracted using Miniprep plasmid extraction kit (Qiagen).

For the expression and purification of P116 protein fragment, the 609 bp fragment was cloned in pQE-30 vector with *Bam *HI and *Pst *I restriction sites. The ligated plasmid DNA was used to transform competent M-15 cells. Transformants were selected on ampicillin (100 μg/ml) and kanamycin (25 μg/ml) plate. M-15 cells containing the recombinant plasmids were cultivated in 5 ml of LB broth and the protein expression was induced by the addition of 1 mM IPTG final concentration, followed by 3 hrs of shaking at 37°C. Bacterial pellet was subjected to SDS-PAGE and western blotting using anti-His antibody to analyze the expression of recombinant protein.

### SDS-PAGE and western blotting

To analyse the expression of the (P116_(N-27)_) recombinant protein, induced and un-induced *E. coli *pellets from 1 ml of grown cultures were resuspended in 100 μl of SDS sample buffer (62.5 mM Tris-HCl, pH 6.8, 10% glycerol, 2.3% w/v SDS, 5% v/v β-mercaptoethanol and 0.05% w/v bromophenol blue) and boiled for 5 min. The proteins were resolved on 10% SDS-PAGE gel and stained with Coomassie brilliant blue R-250. For immunoblotting, after separating proteins on gel, the resolved proteins were transferred onto a nitrocellulose membrane (Sigma-Aldrich) in a transblot apparatus (Mini-PROTEAN III, Bio-Rad, USA). The membrane was blocked in blocking buffer (5% skimmed milk in PBS-T) for 2 h. The blots were washed and incubated with anti-His primary antibody (1:10,000 dilution) or anti-*M. pneumoniae *antibody (1:5,000 dilution) or sera from *M. pneumoniae *infected patients (1:50) for 1 h. Later the blots after washing, were incubated with secondary antibody (1:2000 dilution of anti-mouse, anti-rabbit or with 1:5000 dilution of anti-human antibody conjugated with horse-raddish peroxidase. The blots then developed with 3, 3'-diaminobenzidine tetrabenzidine hydrochloride (DAB)-H_2_O_2 _(Sigma-Aldrich)_._

### Purification and Characterization of N-terminal fragment of P116 protein

Sub-cellular localization studies were carried out to analyse the expression of P116 protein fragment in *E. coli *cells and the protein was found to be expressed in inclusion bodies. For the preparation of inclusion bodies *E. coli*. cells were disrupted by sonication in buffer ((0.05 M Tris (pH 8.0), and 0.3 M NaCl) with 1-min pulses at 1-min intervals 10 times using mini probe (Torbeo, ultrasonic processor 36800-series). The soluble and insoluble fractions were separated by centrifugation at 14,000 rpm at 4°C for 30 min and were analyzed by SDS-PAGE. To purify the protein from the inclusion bodies, *E. coli *pellet from 200 ml of culture was suspended in 1/50 original volume of lysis buffer (0.05 M Tris (pH 8.0), 0.3 M NaCl and 8 M Urea). The lysate was loaded onto a Ni-NTA column (Qiagen) and the bound protein was eluted under denaturing conditions in a buffer containing 0.05 M Tris (pH 8.0), 0.3 M NaCl, 0.3 M imidazole and 8 M Urea. The denatured protein was refolded by dialysis using buffers containing 0.05 M Tris (pH 8.0), 0.3 M NaCl and decreasing concentrations of Urea, 6M-0.5M. The refolded protein was stored in buffer containing 0.05 M Tris (pH 8.0), 0.15 M NaCl, 0.5 M Urea and 5% glycerol. Purified recombinant P116 protein fragment (P116_(N-27)_) was analysed for its reactivity with positive anti-*M. pneumoniae *human sera in western blot assay.

### Expression and purification of the C-terminal recombinant P1 protein fragment P1_(C-40) _of *M. pneumoniae*

Expression and purification of P1 protein fragment was carried out by a protocol described by Chaudhry et al [17].

### Immunization of Rabbits

The protein concentration of both the antigens preparation was determined by Bradford assay. To check the immunogenicity of the two recombinant proteins-P116 and P1, four White New Zealand rabbits (two tests and two controls) were selected. Each one of the two test rabbits was immunized with 300 μl (250 μg) of purified recombinant P116_(N-27) _or P1_(C-40) _protein emulsified in 300 μl Complete Freund adjuvant (CFA, Sigma-Aldrich) intramuscularly (i.m.). Rabbits were subsequently boosted with 300 μl (250 μg) of the same protein in 300 μl Incomplete Freund adjuvant (IFA, Sigma-Aldrich), through the same route on the 21^st ^and 42^nd ^days. Control rabbits were injected with complete and incomplete Freund's adjuvant in normal saline according to the immunization schedule. Blood samples were obtained by ear vein puncture on 0^th^, 14^th^, 21^st^, 28^th^, 35^th^, 49^th ^and 56^th ^days. IgM and IgG antibody responses against the two purified recombinant proteins were analysed by ELISA and end point titres were determined. In order to confirm the specificity of the antisera western blotting with whole *M. pneumoniae *as antigen was also performed.

### Comparative ELISA with purified recombinant P116_(N-27), _P1_(C-40) _and commercial IgM assay

Sera obtained from sixty two patients suffering from respiratory tract infections were analysed for the presence of anti-*M. pneumoniae *IgM antibody (which is an indicator of the acute infection), using Serion ELISA kit. Among the sixty two sera samples tested, thirty one samples were found positive for the anti-*M. pneumoniae *IgM antibodies. We next compared the antibody response seen with Serion kit with the response seen with recombinant P116_(N-27)_, P1_(C-40) _or P116_(N-27) _+ P1_(C-40) _together for IgM antibodies. The experiment was done in duplicate. Briefly, 100 ng of P116_(N-27) _or P1_(C-40) _was added to each well of 96-well microtiter plates. In case of P116_(N-27) _+ P1_(C-40) _assay, P116_(N-27) _and P1_(C-40) _antigens were mixed in 1:1 ratio. The plates were incubated overnight at 4°C and further for one hour at 37°C next day. The plates were washed with PBS-T and blocked with 5% BSA in PBS for 2 h. The plates were subsequently washed twice with PBS-T, once with PBS and incubated with 1:50 diluted patient sera at 37°C for 1 h. The positive control (individual serum, tested positive by the commercial kit) and negative control (individual serum, tested negative by the commercial kit) were included in each ELISA assay and they were also diluted to the same extent. The wells were washed and incubated with HRP-conjugated goat anti-human IgM (Sigma-Aldrich) diluted 1:3000 in PBS-T, for 1 h. The enzyme reaction was developed by the addition of the substrate *ortho-*phenylenediamine (Sigma) (1 mg/ml) diluted in phosphate-citrate buffer (pH 5.0) containing 0.03% (v/v) hydrogen peroxide. The enzymatic reaction was stopped with 100 μl of 2N H_2_SO_4 _and absorbance was read at 490_nm _with ELISA (Bio-Tek Microplate) reader.

### Statistical Analysis

Comparative statistical analysis of ELISA assays for P116 and P1 proteins was done taking Serion IgM kit (100% sensitive and 75% specific as per the manufacturer's claim) as reference test. Analysis was performed with the SPSS version 15.0(Chicago, USA). The parameters of in house ELISA assays were calculated by using Epitable module of Epiinfo (6.04D) software. Cohen's Kappa test was used to find the agreement between two different modalities. The chi-square test of proportions was applied to compare two proportions with the p value < 0.05 considered statistically significant. Cut-off values were calculated by taking median values of the controls.

## Results

### Cloning and Expression of N-terminal fragment of *M. pneumoniae *P116 protein

**Figure 1:**
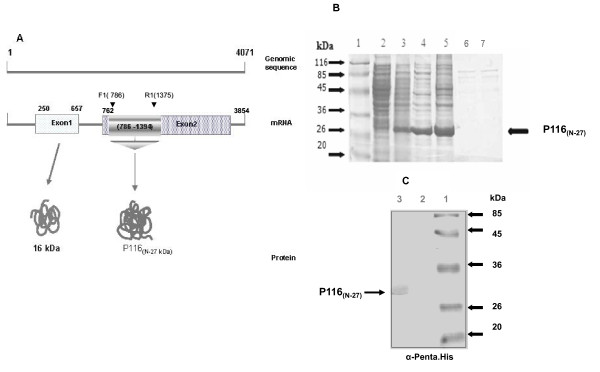
**Cloning and Expression of N-terminal fragment (203aa) of P116 protein of *M. pneumoniae***. **A**: Schematic diagram showing P116 gene sequence and location of fragment of P116 gene selected for cloning and expression. Fig. also shows positions of F1 (forward) and R1 (reverse) primers within the gene coding for P116 protein. **B: **SDS-PAGE analysis of N-terminal P116 protein showing its expression in *E. coli *and its subcellular localization. Lane1: Prestained standard protein marker, Lane 2: Protein extracts of uninduced *E. coli*, Lane 3: Protein extracts of induced (~27 KDa) *E. coli*, Lanes 4&5: *E. coli *pellets of the expressed protein after sonication, Lanes 6&7: Supernatant after sonication. **C: **Immunoblot analysis of crude P116 protein with anti. His antibodies-Lane1: Prestained standard protein marker, Lane 2: Protein extracts of uninduced *E. coli*, Lane 3: Recombinant protein detected with anti. His antibodies.

A 609 bp fragment of P116 gene (786nt-1394nt) coding for a protein of 203 amino acids was successfully amplified by PCR using the designed primers, and was cloned into pGEM-T easy vector. Figure 1A shows the schematic of P116 gene and the location of the amplified gene fragment. The cloned fragment was sequenced using an automated sequencer and its sequence was similar to the published sequence of the P116 gene of the strain FH [25] used in this study. To express the P116 gene fragment, the PCR amplified fragment was sub-cloned into pQE-30 vector and protein expression was analysed on SDS-PAGE. Sub-cellular localization studies showed that protein of mol. wt. of ~27 kDa was mainly expressed in inclusion bodies (Figure 1B). The expressed protein showed reactivity with anti-Penta. His antibody (Figure 1C) as well as with the anti-*M. pneumoniae *antibody (data not shown).

**Figure 2:**
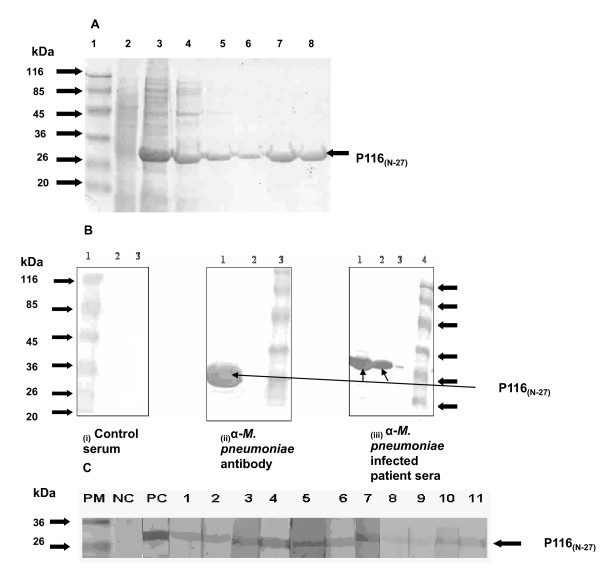
**Purification and Characterization of N-terminal fragment of P116 protein**. **A: **SDS-PAGE analysis showing purification of N-terminal P116 (P116_(N-27)_) protein on Ni-NTA column. Lane 1: Prestained standard protein marker, Lane 2: Protein extracts of uninduced *E. coli*, Lane 3: Protein extracts of induced (~27 KDa) *E. coli*, Lane 4: Flow through, Lanes 5&6: Wash1 & Wash2, Lane 7: Purified P116 protein before dialysis (eluted with buffer containing 8M Urea), Lane 8: Purified P116 protein after dialysis against buffer containing 0.5M Urea. **B: **Immunoblot analysis of purified P116_(N-27) _protein with-**(i) **Healthy control human serum-Lane 1: Prestained standard protein marker, Lane 2: Protein extracts of uninduced *E. coli*, Lane 3: Recombinant protein detected with representative healthy human serum. **(ii) ***M. pneumoniae *antibodies-Lane 1: Recombinant protein detected using rabbit anti-*M. pneumoniae *serum Lane 2: Protein extracts of uninduced *E. coli*, Lane 3: Prestained standard protein marker. **(iii) ***M. pneumoniae *infected patient sera-Lanes 1&2: Recombinant protein detected using *M. pneumoniae *infected patient sera, Lane 3: Protein extracts of uninduced *E. coli*, Lane 4: Prestained standard protein marker. **C: **Immunoblot analysis of purified P116_(N-27) _protein with patient sera infected with *M. pneumoniae *PM: Prestained standard protein marker, NC**: **Negative control (patient sample which tested negative with the reference test) and PC: positive control (patient sample which tested positive with the reference test), Lanes 1-11: patient sera infected with *M. pneumoniae*.

P116_(N-27) _protein was purified under denaturing conditions on a Ni-NTA column (Figure 2A). The yield of the protein was ~5 mg/l. Fractions containing the denatured proteins were pooled in a dialysis bag and the protein was dialysed in buffers, 0.05 M Tris (pH 8.0), 0.3 M NaCl containing decreasing concentrations of Urea, 6M-0.5M at 4°C. Some amount of protein got precipitated after dialysis and the final yield of the refolded protein was ~2 mg/l of the culture (Figure 2A).

### Immunoblot analysis of recombinant P116_(N-27) _with *M. pneumoniae *infected patient sera

Immunoblot analysis was carried out using rabbit anti-*M. pneumoniae *serum (Figure 2B (ii) and sera from *M. pneumoniae *infected patient (Figure 2B (iii). As shown in Figure 2B, P116_(N-27) _was recognized by both the sera strongly. This reactivity was specific as sera from healthy humans (age matched persons without any history of the respiratory tract infections) failed to recognize any such protein (Figure 2B(i)). We next analysed the reactivity of recombinant P116_(N-27) _protein with eleven sera from different patients infected with *M. pneumoniae *and six uninfected patients sera (negative controls), for IgM antibody. All the eleven sera showed reactivity with the P116_(N-27) _protein, while sera from uninfected patients failed to recognize the P116_(N-27)_. These results suggested that the N-terminal region (27 kDa) is one of the immunodominant region of the P116 protein.

### P116_(N-27) _and P1_(C-40) _proteins are immunogenic

**Figure 3:**
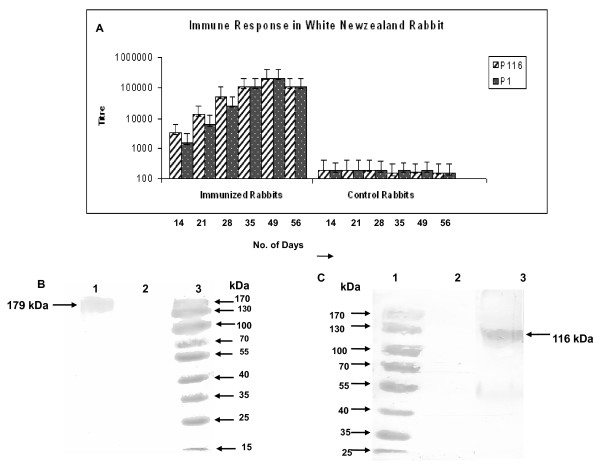
**Immunogenicity and specificity of P116_(N-27) _and P1_(C-40) _proteins and their antibodies**. **A**. Bar diagram showing Immune response in two different White NewZealand rabbits immunized with purified recombinant P116_(N-27) _and P1_(C-40) _proteins. Control rabbits were injected with complete and incomplete Freund's adjuvant in normal saline according to the immunization schedule. **B**. Immunoblot analysis of *M. pneumoniae *lysate showing reactivity of P1(179 kDa) with anti-P1 serum raised in rabbit-Lane1: *M. pneumoniae *lysate, Lane 2: Blank, Lane 3: Prestained standard protein marker. **C**. Immunoblot analysis of *M. pneumoniae *lysate showing reactivity of P116(116 kDa) with anti-P116 serum raised in rabbit-Lane 1: Prestained standard protein marker, Lane 2: Blank, Lane 3: *M. pneumoniae *lysate

To know whether the two proteins P116_(N-27) _and P1_(C-40) _are immunogenic in nature, we formulated these recombinant proteins with CFA and injected them in rabbits. High antibody response was generated against the corresponding protein. The time course of the immune response for each of the recombinant proteins (Figure 3A) showed that antibody titres gradually increased after first and second boost and peaked after the second boost. The end point titres for P116_(N-27) _and P1_(C-40) _were > 2,56,000 for both the proteins. Western blotting with whole *M. pneumoniae *as antigen confirmed the specificity of the antisera raised as they recognized single specific bands for each antigen without any cross-reactivity (Figure 3B &3C).

### Comparative ELISA analysis

**Figure 4:**
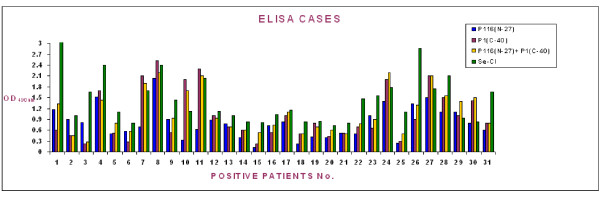
**Comparative ELISA analysis of recombinant proteins with commercial assay with patient sera infected with *M. Pneumoniae***. Comparative ELISA analysis of purified recombinant P116_(N-27)_, P1_(C-40) _and P116_(N-27) _+ P1_(C-40) _with commercial ELISA for IgM antibodies to *M. pneumoniae *with 31 positive patient sera. For each serum the "blue square" shows reactivity with the P116_(N-27)_, whereas "purple, yellow and green squares" show reactivity with the P1_(C-40), _P116_(N-27) _+ P1_(C-40) _and commercial ELISA kit respectively.

A comparative evaluation of the reactivity of the patient sera with P116_(N-27) _and P1_(C-40) _proteins along with Serion (Virion-Serion, GmbH, Germany) kit was carried out to determine immunodiagnostic potential of these recombinant antigens in immunodiagnosis of *M. pneumoniae *infections. A total of 62 sera were analysed for their reactivity to the recombinant proteins individually and together in ELISA assays. In the beginning, these sera were also tested with the commercial Serion kit and thirty one sera were found positive for IgM antibodies to *M. pneumoniae*. Although most patient sera shown to be positive for *M. pneumoniae *antibodies by Serion kit were also positive for their reactivity towards P116_(N-27) _and P1_(C-40) _proteins, however few differences were also observed. Of the 31 positive sera selected based on the reactivity with commercial kit, 28, 27 and 30 sera reacted with P116_(N-27) _and P1_(C-40)_, and P116_(N-27) _+ P1_(C-40) _ELISA assay respectively (Figure 4). Corresponding respiratory samples for eleven of the sixty two sera were also positive by the PCR for 543 bp fragment of *M. pneumoniae *P1 gene.

**Figure 5:**
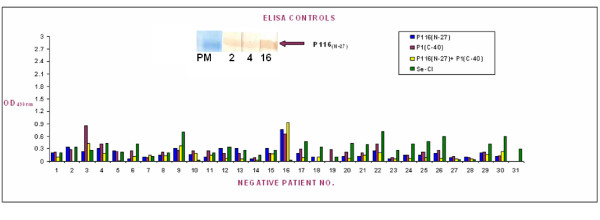
**Comparative ELISA analysis of recombinant proteins with commercial assay with patient sera negative for *M. pneumoniae *infection**. Comparative ELISA analysis of purified recombinant P116_(N-27)_, P1_(C-40) _and P116_(N-27) _+ P1_(C-40) _with commercial ELISA for IgM antibodies to *M. pneumoniae *with 31 negative patient sera. For each serum the "blue square" shows reactivity with the P116_(N-27)_, whereas "purple, yellow and green squares" show reactivity with the P1_(C-40), _P116_(N-27) _+ P1_(C-40) _and commercial ELISA kit respectively (Inset shows western blot results for the three patients negative by commercial ELISA, but positive in P116_(N-27)_, or P1_(C-40) _or P116_(N-27)_+ P1_(C-40) _ELISAs as well as in western blot with P116_(N-27)_).

Interestingly, four out of 31 seronegative patients as determined by Serion kit showed reactivity with the P116_(N-27) _protein or P116_(N-27) _and P1_(C-40) _combined (Figure 5). Western blot analysis for these four cases further confirmed the reactivity of three of the four sera with P116_(N-27) _protein. Western blot could not be performed for one serum sample due to its inadequate volume. Among the seropositive group, 3 sera were negative by P116_(N-27) _based ELISA. Of these 3, one was also negative by P1_(C-40) _based assay. However, all these 3 sera were positive in P116_(N-27) _+ P1_(C-40) _based ELISA.

### Statistical analysis

A comparative analysis of the P116_(N-27), _P1_(C-40)_, P116_(N-27) _+ P1_(C-40) _ELISA and assay with Serion kit (the reference test), was done (Table 1). The cut off value was taken as an absorbance 0.3. At 0.3 absorbance, the senstivity and specificity was 90.3% and 87.0% for P116_(N-27), _87.1% and 87.1% for P1_(C-40)_, and 96.8% and 90.3% for (P116_(N-27) _+ P1_(C-40)_) respectively. The p value for all the three assays was found to be < 0.001, which is highly significant and there was a good correlation and association between them. There was an observed agreement of 77.4% and 74% between P116_(N-27), _P1_(C-40) _and commercial ELISA respectively and is the highest (87%) between the P116_(N-27) _+ P1_(C-40) _combined ELISA and commercial ELISA.

**Table 1:** Representation of statistical parameters of comparative ELISA analysis for IgM antibodies to *M. pneumoniae* -

Parameters	P116_(N-27)_	P1_(C-40)_	P116_(N-27) _+ P1_(C-40)_
**Cut Off Value (OD_490_)**	0.30	0.30	0.30
**Sensitivity**	90.3%	87.1%	96.8%
**Specificity**	87.0%	87.1%	90.3%
**Positive predictive value**	87.5%	87.1%	90.9%
**Negative predictive value**	90.0%	87.1%	96.6%

## Discussion

Laboratory investigations are important in the diagnosis of *M. pneumoniae *infections, as it is often difficult to differentiate different etiological agents by clinical symptoms. Even though a number of clinical tests such as PCR have been developed and applied to identify *M. pneumoniae *infections, serology is probably the most frequently used method to diagnose *M. pneumoniae *infections as PCR based tests are expensive and require specialized equipment [28]. Serological testing is often hampered by inter-species cross-reactions and even non-specific reactions [30] as the currently available serological tests are based on crude cellular fractions of *M. pneumoniae*. An ELISA employing purified *M. pneumoniae *surface proteins would be having inherent advantage of specificity. One of the major difficulties of developing serological test for the detection of *M. pneumoniae *is the difficulty to express its proteins in heterologous expression system as the Mycoplasmas use the UGA opal codon to incorporate tryptophan rather than as a stop codon as in the universal genetic code [31], leading to premature termination of the protein synthesis. Although, a few reports from the western world are available, no study has been done in the Indian population, which investigates the immunodiagnostic potential of the *M. pneumoniae *116-kDa virulence factor (P116). We have been systematically analysing the serodiagnostic potential of various surface adhesion antigens of *M. pneumoniae *and previously showed that a C-terminal fragment of P1 and P30 protein hold potential to be used for the diagnosis of *M. pneumoniae *infections [17,19].

In furtherance to these studies, we expressed an N-terminal fragment of P116 protein [28] using an *E. coli *expression system with an N-terminal 6His-tag. The expressed protein was ~27 kDa in size and was purified on a Ni-NTA column under denaturation conditions. The yield of the protein was ~5 mg/l. A simple refolding protocol was developed to get the protein in soluble form. Even though the protein was expressed in inclusion bodies, a significant yield of the protein after refolding (~2 mg/l) could be obtained. The refolded soluble protein was recognized by anti-*M. pneumoniae *antibodies, by most of the *M. pneumoniae *infected patient sera and also by experimentally infected rabbit sera by immunoblot analysis. Our results were in agreement with a previous lone study by Duffy et al who expressed GST fusion protein fragments of P116 protein and showed that a 53 kDa P116 protein fragment (pGEX-3X-MP661) encompassing 9aa-474aa was recognized by most of the patient sera in ELISA [28]. The P116 fragment expressed in this study represented a segment of pGEX-3X-MP661 protein and was also expressed without a long fusion tag GST. The recombinant P116_(N-27) _was also found to be immunogenic in rabbit. The gene sequence of the P116_(N-27) _fragment was found similar to the sequence of P116 gene of *M. pneumoniae *strains FH and M-129 given in the database. The nucleotide sequence of the gene encoding the 116 kDa protein, and consequently also the epitopes of the protein, is highly conserved between *M. pneumoniae *M-129 and FH [28], which are representatives of the two *M. pneumoniae *groups. To know about the serodiagnostic potential of P116_(N-27) _IgM ELISA, sixty two Indian patients suffering with *M. pneumoniae *infections were analyzed. All these patients sera were also analyzed by the commercially available Serion (Virion-Serion, GmbH, Germany) kit. In comparison to the reference kit, which is reported to be 100% sensitive and 75% specific, ELISA assay based on purified P116_(N-27), _P1_(C-40) _and (P116_(N-27) _+ P1_(C-40)_) proteins showed 90.3%, 87.1% and 96.8% sensitivity and 87.0%, 87.1% and 90.3% specificity respectively. The p value for all the three assays was found to be < 0.001, which is highly significant and there was a good correlation and association between them. Our results also demonstrated that an ELISA assay based on two recombinant proteins was more sensitive and specific. During the course of this study, a report by Drasbek et al described the utility of P116 and P1 recombinant fragments for correct diagnosis of atypical pneumonia caused by *M. pneumoniae *[32]. The difference between these two studies is that we used shorter fragments of P116 and P1 proteins to take care of the specificity problems. We could achieve moderate level of expression for both these recombinant proteins (2-4 mg/l of culture) in the present study. One of the major requirements with regard to the development of an ELISA test for detecting *M. pneumoniae *infection is to avoid possible cross-reactions with antibodies to *M. genitalium *[33]. To overcome this cross-reactivity, use of synthetic peptides has been tried instead of whole protein preparations [34]. We could not found significant sequence similarity when we aligned the sequence of P116_(N-27) _protein of *M. pneumoniae *with *M. genitalium*. Thus, our study using the short recombinant fragment of P116 and P1 proteins together is an important step towards developing *M. pneumoniae *specific and sensitive diagnostic assay. Importantly, since present study was conducted in a geographically distinct location than the study by Drasbek et al, the study provides an important confirmation of the global applicability of the findings of Drasbek et al. Together these limited trials also indicate that recombinant P116 and P1 proteins can be useful as serodiagnostic agents.

## Conclusions

The present study describes a simplified approach for the large-scale production of an immunodominant fragment of P116 protein of *M. pneumoniae*. The report further describes the immunodiagnostic potential of P116 and P1 proteins individually or combined by ELISA on parallel patient's serum samples. Since both protein fragments are also immunogenic, it will be worth-while also to look for vaccine potential of these recombinant proteins. Surveillance or reporting system for *M. pneumoniae *infections eg. positive serological results for *M. pneumoniae *based on rapid and specific diagnostic tests, would be useful for physicians in understanding the regional epidemiology of *M. pneumoniae *infections, and also decide the empirical antibiotic treatment to be used in these cases.

## Competing interests

The authors declare that they have no competing interests.

## Authors' contributions

This work is a part of Ph.D. thesis of IT, under supervision of RC, Professor, Deptt. of Microbiology, All India Institute of Medical Sciences, New Delhi, India. RC and PM participated in the study design and coordination. RC provided the reagents and other facilities. PM designed the primers and provided technical guidance. Both of them edited the manuscript. BK performed western blotting with *M. pneumoniae *lysate. All authors read and approved the final manuscript.

## Pre-publication history

The pre-publication history for this paper can be accessed here:

http://www.biomedcentral.com/1471-2334/10/350/prepub
